# Letter about ‘“Here today, gone tomorrow” – What happened to recurrent brief depression?”

**DOI:** 10.1017/S0033291726103523

**Published:** 2026-02-27

**Authors:** Mauro Giovanni Carta

**Affiliations:** University of Cagliari: Universita degli Studi Di Cagliari, Italy

Dear Editor,

I read with great interest the recent commentary by Nestorovic and Baldwin, ‘“Here today, gone tomorrow” – What happened to recurrent brief depression?’, published in *Psychological Medicine* (Nestorovic & Baldwin, 2026). The authors persuasively argue that research into recurrent brief depression (RBD) has slowed despite its potential clinical importance (Angst, Kasper, & Weiller, 2000; Baldwin, 2003), particularly for young people. I would like to add evidence from our previous work that supports and may extend their conclusions.

A decisive turning point in the apparent epidemiological disappearance of RBD occurred when the Composite International Diagnostic Interview (CIDI), employed in World Mental Health Surveys (Kessler & Ustün, 2004), implemented a diagnostic algorithm for depressive disorders that excluded all individuals who had not experienced depressive symptoms for at least 14 consecutive days. As a result, individuals eperiencing recurrent brief depressive episodes in the absence of major depressive disorder were systematically screened out from case identification. This methodological choice has likely contributed to the underrecognition of RBD in epidemiological research, despite its clinical significance and association with suicide risk. I participated in translating the instrument into Italian and reported this inconsistency to Jules Angst and to members of the WHO research group; however, since the research was already at an advanced stage, and the algorithm could not be changed, I subsequently left the Italian group.

The immense amount of data drawn from the World Mental Health Surveys, which led to numerous scientific publications forming the basis of subsequent knowledge about depressive disorders, therefore, contained no information about RBD. Furthermore, as regards mood disorders, the instrument itself had obvious limitations, and even attempts to justify its validity (Kessel et al., Kessler et al., 2006), were saddled with criticism and seem to have done little to resolve any doubts (Carta, Hardoy, C, & Fryers, 2008).

Today, contradictions are emerging on certain controversial issues, such as the so-called suicide paradox, namely that suicide mortality is higher in men despite the greater prevalence of major depressive disorder in women, perhaps fueled by the cited biases in epidemiology surveys. The well-documented elevated suicide risk associated with RBD, together with evidence suggesting that RBD is more frequent in males, may contribute to explaining this paradox. If depressive morbidity in men is more likely to manifest through brief, recurrent, rhythm-dysregulated episodes rather than through syndromal major depressive disorder, epidemiological strategies relying primarily on episode-duration thresholds may underestimate clinically significant depressive pathology in males and its associated suicide risk. Nestorovic and Baldwin’s paper cites our research (Carta et al., 2003) showing a higher frequency of RBD in young people, whereas suicide is more common among the elderly. However, this evidence does not contradict the hypothesis (in agreement with data from the Zurich cohort) that those with RBD in youth (frequently males) are more likely to develop RBD + Major Depressive Disorder (MDD) in old age, a rare subtype of MDD more common in males, but with a very high suicide risk.

Our considerations directly align with the concerns raised by Nestorovic and Baldwin. They support the notion that an exclusive emphasis on episode duration risks the underrecognition of clinically significant depression in young people, particularly in males. They also reinforce the hypothesis that RBD may lie at the interface between unipolar and bipolar spectrum disorders, consistent with its associations with affective temperaments and dysregulation of biological and social rhythms.

I therefore fully endorse the authors’ call for renewed research efforts into RBD. Future studies should explicitly consider its relationship with mood spectrum conditions, include longitudinal community samples of adolescents and young adults, and focus on outcomes such as recurrence, functional impairment, and suicide risk, with attention to potential sex-specific patterns of presentation.

Recurrent brief depression may indeed be ‘here today, gone tomorrow’ at the symptom level – but it should no longer be absent from the research agenda.
